# A Case of Renal Pelvic Cancer Complicated by Horseshoe Kidney Treated with RoboSurgeon Gasless Single-Port Retroperitoneoscopic Nephroureterectomy

**DOI:** 10.1155/2018/3231480

**Published:** 2018-09-18

**Authors:** Hiroaki Suzuki, Kosuke Takemura, Kazumasa Sakamoto, Madoka Kataoka, Masaya Ito, Yasukazu Nakanishi, Ken-ichi Tobisu, Fumitaka Koga

**Affiliations:** Department of Urology, Tokyo Metropolitan Cancer and Infectious Diseases Center Komagome Hospital, Japan

## Abstract

A 78-year-old man who had a horseshoe kidney (HSK) visited us because of gross hematuria and suspicious urine cytology. CT and MRI revealed a right renal pelvic tumor of 28 mm in diameter. He underwent gasless single-port retroperitoneoscopic nephroureterectomy with division of the isthmus via the right pararectal small incision using the RoboSurgeon system. Pathological diagnosis was invasive urothelial carcinoma, grade 3, pT3, pN0, LVI0, RM0. He was discharged from hospital on the 6th postoperative day without any perioperative complication. He has no evidence of disease clinically and radiologically 19 months after the operation. Only a few cases of upper tract urothelial carcinoma complicated by HSK treated with minimally invasive surgery have been reported in English literature. This is the first case successfully managed with RoboSurgeon gasless single-port retroperitoneoscopic nephroureterectomy.

## 1. Introduction

Horseshoe kidney (HSK) is a relatively common kidney malformation accounting for approximately 0.15-0.25% of the population [1]. However, upper tract urothelial carcinoma (UC) complicated by HSK is a rare clinical condition in daily urology practice. Because of complicated anatomical features of HSK, radical nephroureterectomy (RNU) via minimally invasive approaches would be challenging. We report the first case of HSK with upper tract UC, successfully managed with RoboSurgeon gasless single-port retroperitoneoscopic RNU [2, 3].

## 2. Case Presentation

A 78-year-old man with HSK visited our hospital due to asymptomatic gross hematuria and renal hypofunction in August 2016. He had comorbidities of Charlson index of 4, including chronic kidney disease (CKD; creatinine 1.71 mg/dL, estimated glomerular filtration rate [eGFR] 31 mL/min/1.73m^2^), atrial fibrillation requiring warfarinization, and type 2 diabetes (HbA1c 6.7%). He had undergone coronary artery bypass grafting at his age of 69 years.

**Figure 1:**
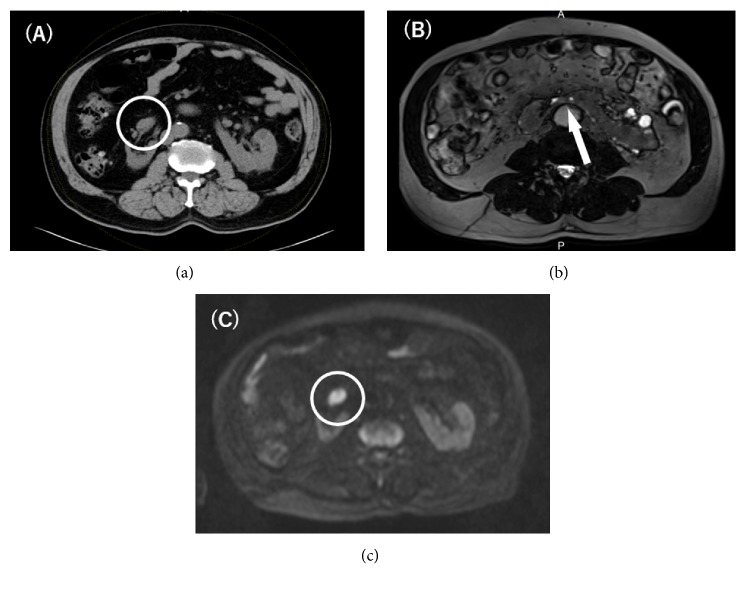
(a) CT scan axial image. A 28 mm tumor is shown in the right renal pelvis. (b) T2-weighted MR image.* Arrow* indicates the isthmus of a horseshoe kidney. (c) Diffusion-weighted MR image. The tumor in the right renal pelvis shows high signal.

Blood tests did not show any abnormalities other than elevated serum creatinine. Urinalysis and urinary sediments revealed proteinuria (2+) and hematuria (erythrocyte count 50-99/HPF). Voided urine cytology was suspicious for UC. Computed tomography (CT) and magnetic resonance imaging (MRI) demonstrated an atrophic right renal unit and a right renal pelvic tumor of 28 mm, which exhibited high signal intensity on diffusion-weighted MRI (Figure 1). Extrapelvic extention of the tumor, lymph node swelling, or distant metastasis were not evident. Only one renal artery was identified for each renal unit on MRI. Cystoscopy revealed no bladder tumor.

**Figure 2:**
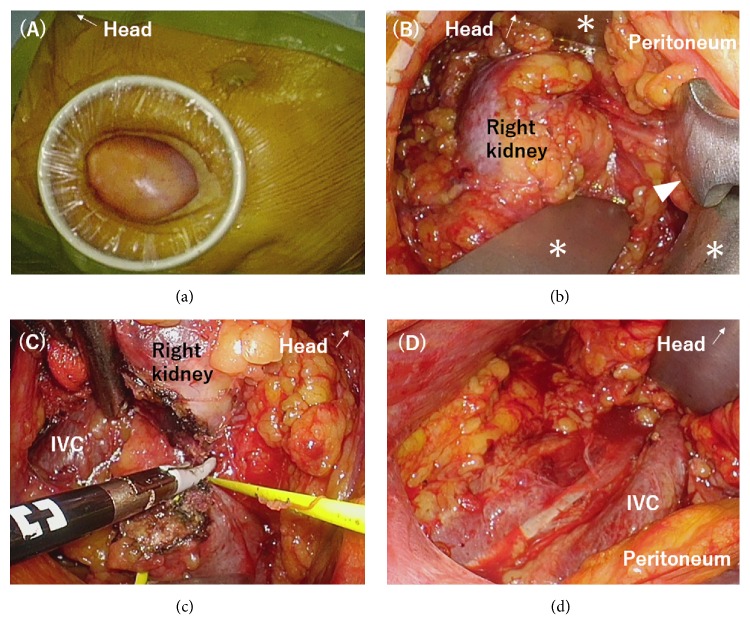
(a) A wound retractor was placed at the pararectal incision of 6 cm made in the right upper quadrant. (b) Nephroureterectomy was carried out by keeping the retroperitoneal working space made along the anatomical plane using the Omni-Tract retractor system and PLES retractors (an arrow head). Asterisks indicate blades of the Omni-Tract retractor system. (c) Isthmusectomy was conducted using a bipolar sealing device. A vessel loop was passed beneath the isthmus. (d) The right retroperitoneal after completion of right nephroureterectomy and isthmusectomy. A drain tube was placed here after extensive saline irrigation.

Under the clinical diagnosis of UC complicated with HSK (cT2 or less N0M0), the patient underwent RoboSurgeon gasless single-port retroperitoneoscopic right RNU with isthmusectomy in December 2016. In the RoboSurgeon system, intracorporeal surgical procedures are conducted under the three-dimensional (3D) magnified vision using Endoeye flex 3D deflectable videoscope (Olympus, Tokyo, Japan) and a high-definition 3D organic electroluminescent head-mounted display (Sony Corporation, Tokyo, Japan). Briefly, a pararectal incision of 6 cm was made in the right upper quadrant. The surgical plane between the transverse fascia and paraperitoneal fat was developed to make a wide working space extraperitoneally. Alexis Wound Retractor (Applied Medical, California, USA) was placed to make a single port of 5.5 cm in diameter (Figure 2(a)), and Omni-Tract FastSystem (Integra, Pennsylvania, USA) and PLES retractors (Innomedics Medical Instruments Inc., Tokyo, Japan) were used to keep an extraperitoneal working space (Figure 2(b)). The lateroconal fascia was incised to develop the Gerota's fascia anteriorly and to identify the isthmus and inferior vena cava (IVC). The fibrofatty tissue was dissected from the IVC, and small branches from the IVC and aorta were divided using clips and LigaSure (Covidien, Minnesota, USA). Finally, main branches of the right renal artery and the right renal vein were ligated and divided. The right ureter was then clipped to shut the urinary flow off. Then, the right kidney was completely mobilized, preserving the right adrenal gland. Isthmusectomy was conducted using Sonicision (Covidien, Minnesota, USA) and LigaSure (Figure 2(c)). The right ureter was dissected to the ureterovesical junction and RNU with bladder cuff excision being completed. The bladder defect was closed with absorbable sutures and water-tightness was confirmed. The operative field was irrigated with saline of 2L, a drain tube was placed in the right retroperitoneal space (Figure 2(d)), and the wound was closed. The operation time and blood loss were 304 minutes and 510 mL, respectively.

**Figure 3:**
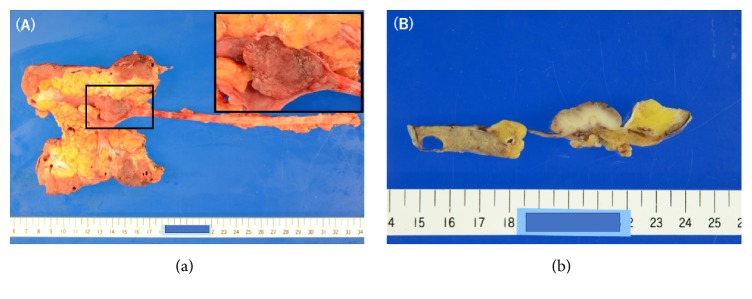
(a) A nodular pedunculated tumor measuring 3.2*∗*2.4*∗*1.0 cm in the right renal pelvis. Inset, magnified image of the tumor. (b) Gross appearance of formalin-fixed tumor section. Surgical margin was negative microscopically.

Gross appearance of the resected specimen showed a nodular pedunculated tumor measuring 3.2*∗*2.4*∗*1.0 cm in the right renal pelvis (Figure 3). Specimen weight was 162 g and the size of the kidney part of the specimen was 11.0*∗*5.0*∗*3.5 cm. The pathological diagnosis was high grade (grade 3) invasive UC with parapelvic fat microinvasion (pT3). No lymph node metastasis (pN0, 0/1), lymphovascular invasion, or positive surgical margin was noted.

The patient resumed diet intake and ambulance on the first postoperative day, and the drain tube and the urinary catheter were removed on the 4th postoperative day. He left the hospital on the 6th postoperative day without any postoperative complication. He did not select any adjuvant therapies. He is free of the disease 19 months after RNU and his serum creatinine level is 1.92 mg/dL with eGFR of 27.0 mL/min/1.73m^2^.

## 3. Discussion

HSK is the most common kidney malformations occurring in approximately 0.15-0.25% of the population [1]. As one of the anatomical features, there are aberrant blood vessels in the isthmus with a possibility of 60% [4]. The aberrant blood vessels bifurcate from the aorta, the common iliac artery, the internal and external iliac artery, the inferior mesenteric artery, or the sacral artery [5]. As minimally invasive surgery on HSK, laparoscopic pyeloplasty or isthmusectomy to benign disease is often reported in recent years. However, RNU via minimally invasive approaches for cases of UC has been seldom reported. Minimally invasive RNU is challenging because of difficulty to identify and manage to transect multiple renal arteries and veins and to perform isthmusectomy. Therefore, in many cases, open surgery tends to be selected.

To our knowledge, there are two reports of RNU via minimally invasive approaches to UC complicated with HSK by searching in Pubmed [6, 7] (Table 1); transperitoneal laparoscopic and hand-assisted laparoscopic approaches were utilized, respectively. Authors of these case reports mentioned that it is important to preoperatively evaluate anatomy of major renal vessels using 3D-CT and to pay attention to aberrant small renal vessels that could not be identified on imaging studies. In this respect, laparoscopic or retroperitoneoscopic surgery is advantageous because of using magnified vision.

We believe that correct surgical plane dissection and avoidance of unnecessary destruction of the normal anatomical structures is important to achieve optimal cancer control and minimally surgical invasiveness. In this context, RoboSurgeon gasless single-port retroperitoneoscopic surgery is suitable for urological malignancies arising from retroperitoneal organs [2]; we can avoid unnecessary peritoneal injury and thus postoperative intraabdominal complications. Furthermore, we can avoid intraabdominal tumor dissemination even if tumor cells accidentally spill during operation by extensive saline irrigation.

For the standard RoboSurgeon retroperitoneoscopic RNU, we utilize two ports: the flank and lower abdominal port [8]. In the present case, the operation was done via single-port, anterior extraperitoneal approach through a pararectal small incision, which would be advantageous for the case of HSK; first, it is easier and safer to identify and transect small aberrant renal vessels arising from the aorta, IVC, and common iliac vessels. Second, isthmusectomy can be performed easily and safely just below the single port. Third, the single skin incision which allows extraction of the surgical specimen would be preferable to two incisions of the standard 2-port approach in terms of minimally invasiveness.

In conclusion, this is the first case of HSK with upper tract UC, successfully managed with RoboSurgeon gasless single-port retroperitoneoscopic RNU. This operation could be one of reasonable minimally invasive surgical approaches for upper tract UC patients complicated with HSK.

## Figures and Tables

**Table 1:** 

*Authors (year)*	*Approach*	*Operation time (min)*	*Blood loss (ml)*	*complications*
*Palmer et al. (2011)*	Transperitoneal laparo (HALS)	—	—	None
*Murakami et al. (2007)*	Transperitoneal laparo	300	400	None
*The present case*	Retroperitoneal RoboSurgeon	304	510	None

HALS: Hand-assisted laparoscopic surgery.

## Data Availability

The datasets generated and/or analyzed during the current study are not publicly available due to protection of the patient's privacy but are available from the corresponding author on reasonable request.
